# In the VEST trial: are we missed to address the pathology incurred by the external stent?

**DOI:** 10.1186/s13019-021-01640-6

**Published:** 2021-09-08

**Authors:** Vijayanand Palanisamy, Valikapthalil Mathew Kurian, Rajan sethuratnam

**Affiliations:** Institute of Cardiovascular Diseases, The Madras Medical Mission, 4A, Mogappair, Chennai, Tamilnadu, 600037 India

**Keywords:** Vest trial, Saphenous vein, Coronary artery bypass grafting

## Abstract

In the VEST IV trial, the author concluded that external stenting of saphenous vein graft mitigates its remodeling and also significantly reduces the diffuse intimal hyperplasia and development of lumen irregularities at 4.5 years after coronary artery bypass grafting surgery. We also have valuable a suggestion in addition to external stenting that might nullify the pathology caused by the stent and might enhances graft patency.

## To the Editor,

We have read the VEST IV trial [1] by David P Taggart and the team, with great expectation. We would like to appreciate the author for their effort to strengthen the weak link in coronary artery bypass grafting (CABG). In their study, the author had shown that the external stenting had aided in maintaining great saphenous vein (SVG) lumen uniformity and hindered the intimal hyperplasia at 4.5 years after CABG.

We would like to bring a major pathology incurred by stent for the author’s notice. In VEST IV trial, they have shown that early graft failures in both groups (30% stented versus 23% nonstented SVG, p = 0.42) were comparable. Restrictive annuloplasty for mitral regurgitation would result in functional mitral stenosis/ patient–prosthesis mismatch [2, 3]. Likewise, the valve leaflet coaptation height and the trans-valvular gradient might have increased by deploying the external stent. This might have been led to the accelerated early graft failure in the stented group.

We recommend few modifications in the VEST trial, which will help them, achieve better venous graft patency.

The arterial system is closed, pressured, and with a potentially bidirectional flow. The presence of a valve in the arterialized venous conduit may hinder this functionality. Whitney et al. did a detailed study using time-lapse angiography throughout 11-year and demonstrated that venous valves cause turbulence and dilatation of the vein segment nearer to the valve [4].

With a flow rate of 10–30 ml/min, valves begin to close and the vein valve’s closure creates a pressure trap in the segment distal to the valve. Segmental hypertension results in an accelerated atherosclerotic process in vein grafts [5]. Mills [6] found that venous valves are the potential site for thrombosis with embolization leading to the critical graft disease and named them as “the bad boys”.

A simple valvulotomy may be used to split these valves allowing a more even flow and avoiding the air trapping and thrombus formation in the valve recesses. A potential cause for perioperative infarction or early graft failure may be avoided. The potential risk factor for valvulotomy was intimal injury and residual valve inside the vein segment. Using modern valvotomes, studies show that the intimal injury was only minimal and the remnant valves also get resolved during the due course [7].

A study by Monsefi et al. revealed that patients with valvulotomized SVG (v-SVG) had good clinical outcomes with a patency rate of all v-SVG was 97.1 versus 95.8% of arterial grafts at 18 ± 6 months postoperative period [8]. Intraoperative flow measurements showed a significant increase (20.2 mL/min; p < 0.01) of flow in the venous bypass grafts after valvulotomy [8].

In 2018, Anli et al. revealed that the mid-term patency of v-SVG was 96.1% in comparison with 96.7% for the arterial grafts at 3.1 ± 2 years [9].

Lajos et al. [10] found that the patency of sequential, valveless veins was 88.6% versus 72% for reversed valvular segments (p < 0.01) when analyzed 436 patients during the follow-up for 8–12 years. They named these valveless veins “good veins”. They also added that if good veins were combined with internal mammary arterial graft, the survival of the patient was improved (p < 0.0057).

SVG on average will have 10–12 valves with more valves located in a below-knee position [11]. While performing CABG, a valveless segment of the vein should be preferred. Secondly, if the valve is present then valvulotomy should be done.

It’s evident from the VEST IV trial that intimal hyperplasia was hindered by applying external stents thereby delayed graft failure. By deploying external stents over the vein in addition to valveless vein/v-SVG, we can avoid the potential risk factors for both early and late venous graft failure.

## Data Availability

Nil.

## Abbreviations

CABG: Coronary artery bypass grafting
SVG: Great saphenous vein
v-SVG: Valvulotomized great saphenous vein
